# Supplementary material for: Effective plans for hospital system response to earthquake emergencies
Source: Nat Commun. 2020 Aug 28;11:4325. doi: 10.1038/s41467-020-18072-w (PMC7455727; doi:10.1038/s41467-020-18072-w)
Supplement: Supplementary file 1 — Supplementary Information [file 41467_2020_18072_MOESM1_ESM.pdf]

Effective Plans for Hospital System Response to Earthquake Emergencies

Supplementary Information

Ceferino et al.

Supplementary Note 1

Lima is a fast-growing megacity with a population close to 10 million people.[1] Though the center of the city is denser, the peripheral areas of the city have become heavily populated over the last few decades. Currently, close to three million people live in peripheral zones in slums,[2] where families are low-income, who often start constructing their homes with precarious materials, e.g., wooden shacks, and then upgrade them to confined-masonry buildings over timespans ranging from a few years to decades.[3] Figure 1 shows how heavily populated the peripheries are. The population distribution in this plot represents the average number of people over 24 hours in grids of 1 km^2. Population density is dynamic, but often people spend most time at their residential buildings, mainly during nighttime. Thus, we considered that this average distribution is a reasonable representation of nighttime population densities.

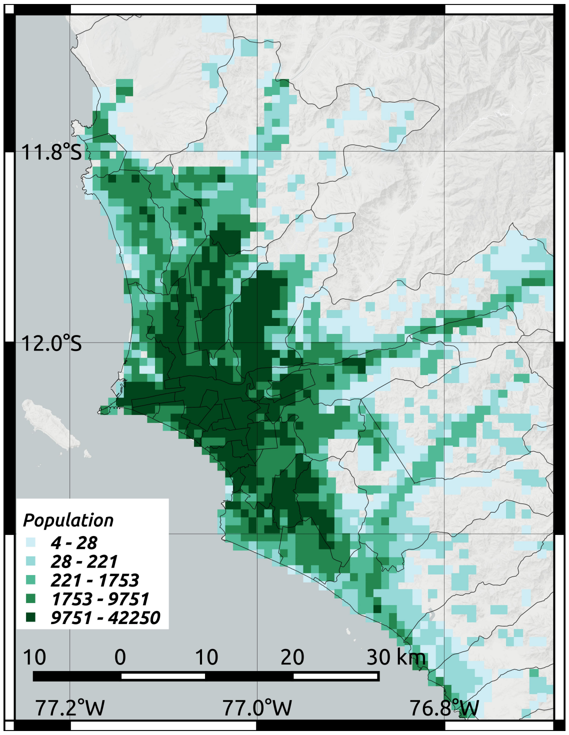

Supplementary Figure 1: Spatial distribution of population density in Lima per km^2. Data obtained from LandScan.[4] The intervals in the two plots represent quintiles on the spatial data. Source data are provided as a Source Data file, and the base map layer is available under a Open Database Licence (©OpenStreetMap Contributors).

The seismic analysis included the assessment of the number of fatalities and injured people with three types of severities caused by an M 8.0 earthquake occurring at nighttime in Lima, when people are often within their houses. As observed in previous earthquakes, the model considers that most casualties are caused by earthquake damage to buildings in the city. Our mean estimates indicate that the M 8.0 earthquake will cause 60.3k people with injuries of severity 1, 18.9k of severity 2, 2.8k of severity 3, and 5.6k immediate fatalities. People with injuries of severity 1 will require basic medical aid and no hospitalization. People with injuries of severity 2 will require hospital treatment, but the injuries are not life-threatening in the short term, and people with severity

3 will require immediate hospitalization otherwise injuries become life-threatening.[5,6] Figures 2a and 2b show the mean spatial distributions of injured people with severity 2 and 3 in the city. Because in the model, casualties are result of building damage, the spatial distribution of patients with severity 2 and 3 are heavily cross-correlated and particularly concentrated in areas with large number of buildings that collapse.

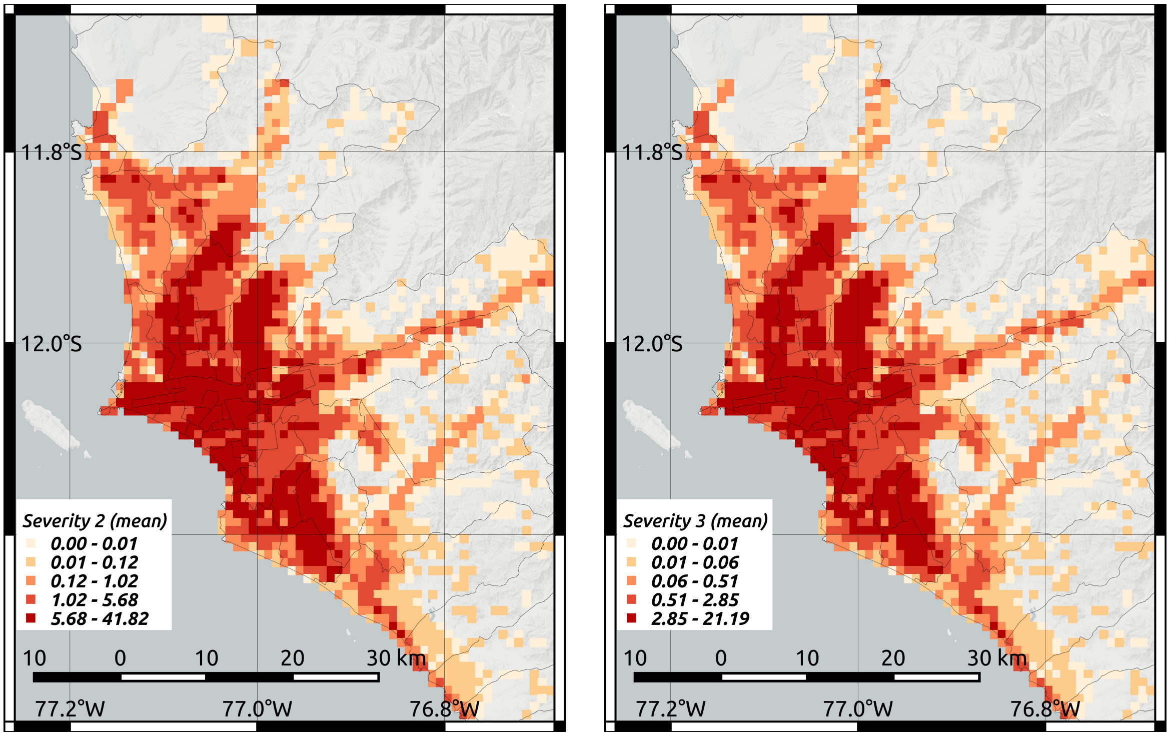

Supplementary Figure 2: Spatial distribution of patients with a) injuries of severity 2 and b) injuries with severity 3. The intervals in the two plots represent quantiles on the spatial data. Source data are provided as a Source Data file, and the base map layer is available under a Open Database Licence (©OpenStreetMap Contributors).

The distributions of casualties shown in Figures 2a and 2b result from the combination of multiple factors, including the vulnerability of the infrastructure and its spatial distribution, the earthquake shaking including soil conditions that can amplify it quite significantly, and the spatial distribution of people at the time of the earthquake (Figure 1). While earthquake shaking and the vulnerability of infrastructure do not change at different times of the day, the spatial distribution of people does, thus the distribution of casualties will be different if the earthquake occurs during the daytime. Our previous studies highlight that it is very important to track such changes at least during other two very distinctive scenarios: working hours and commuting times.[6,7]

As explained in the Methods section, lack of data did not enable us to explicitly model these two scenarios; however, we present Figure 3 to discuss their potential casualty scenarios. The plot shows a normalized spatial distribution of patients who will need surgical procedures. It is estimated as the division between the mean number of patients who will require surgical procedures and the total population at nighttime per km^2. Figure 3 shows that the patient rates in the four centric

districts is close to the median values of the entire city. The average estimate across these four districts is 0.48 patients per thousand people, i.e., 490 patients and ∼1 M people there at the time of the earthquake. Outside these four districts, the rates have large variability. In multiple areas in the periphery, there are high rates, with some hotspots reaching values up to 1 or 2 patients per thousand people. These high rates are a result of significant building vulnerabilities and poor soil conditions in the region, especially in the coastal districts, e.g., softer sands in Villa El Salvador and softer clays in La Punta. Yet, the average rate outside the centric districts is 0.52 patients per thousand people, only 7% larger than in the centric districts.

Assessments aiming to evaluate the surge in casualties during the day can combine the population distribution and building occupancy during such hours with these rates to understand the distributions of healthcare demand and capacity at different times. We expect people to mobilize to the center of the city during the day. While this mobilization might help reduce the number of casualties in the periphery, we do not expect this reduction to solve the mismatch between demands and capacity. If we perform a simple linear extrapolation with the estimated rates, we observe that 40% of the total population in Lima would need to mobilize to the four centric districts during the day so that there is no mismatch between demands and capacity. While lack of data did not enable us to track the increase in density during the daytime in these four districts, we were able to use census information to estimate an upper bound on the number of people in these districts during the daytime. The census information in Lima shows that only 25% of the population outside these centric districts works in different districts from where they live.[8] While not all of these people will leave their districts to go to work to these four centric districts, even if they do, Lima would still face a mismatch between demands and capacities during other times of the day as a result of the heavy concentration of healthcare resources in the center of the city.

The seismic analysis also included the assessment of the ability of hospitals to function after the earthquake. Figure 4 shows the mean functionality ratio of operating rooms in the 41 healthcare campuses that were analyzed. The ratio represents how likely are operating rooms to function after the M 8.0 earthquake considering their structural vulnerabilities, their HSI score, and their proneness to experience large shaking intensities as a result of the proximity to the earthquake fault or the soil conditions. Though the absolute number of functional operating rooms was higher at the center of the city, the spatial patterns of of functionality ratios did show a strong prevalence of high ratios in particular zones of the city. Instead of geographical location, construction year was a better indicator of the hospitals' ability to function, as most newer hospitals showed higher functionality ratios.

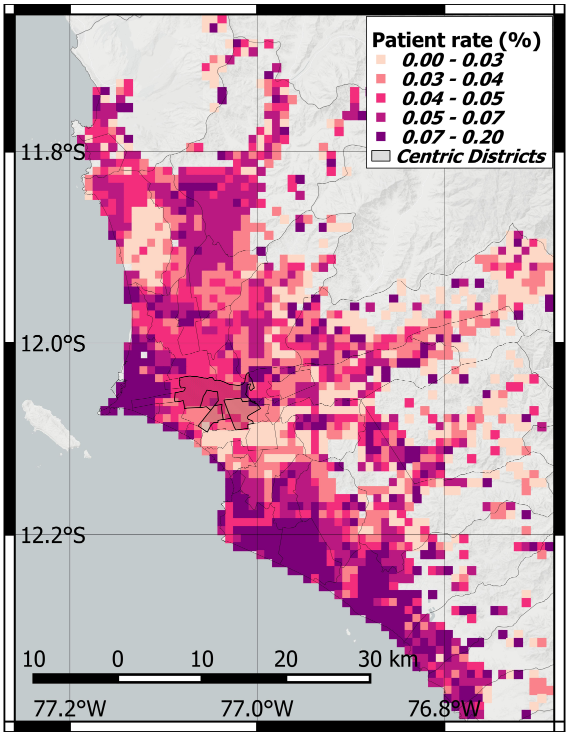

Supplementary Figure 3: Spatial distribution of injury rates estimated as the mean number of earthquake patients who will need surgical procedures and the total number of people per km^2 for the M 8.0 earthquake. The centric districts, Lima, Breña, La Victoria and Jesús María, where the healthcare resources are heavily concentrated. The intervals in the plot represent quantiles (20th-percentile increments) on the spatial data. Source data are provided as a Source Data file, and the base map layer is available under a Open Database Licence (©OpenStreetMap Contributors).

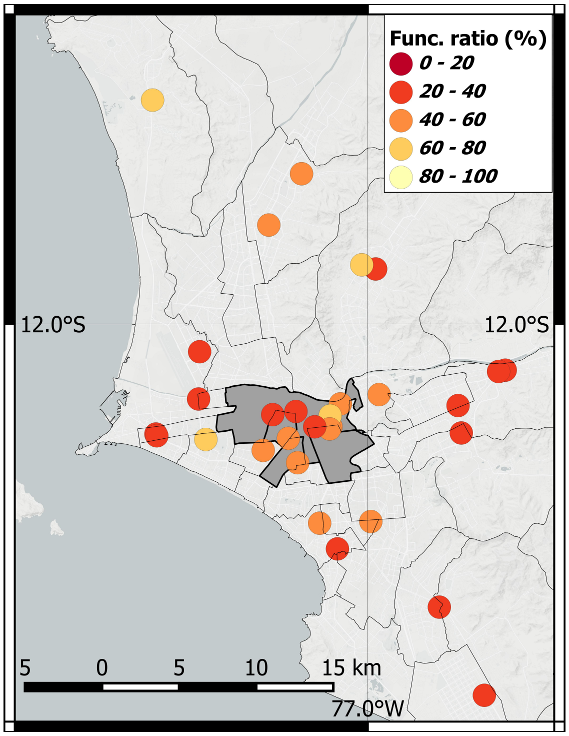

Supplementary Figure 4: Spatial distribution post-earthquake functionality ratio. Newer hospitals, some of the located in the city center, tend to perform better than the older ones. Few hospitals that did not have operating room capacities under normal conditions were assigned 0% functionality ratio. Source data are provided as a Source Data file, and the base map layer is available under a Open Database Licence (©OpenStreetMap Contributors).

Supplementary References

[1] Instituto Nacional de Estadísticas e Informática (INEI). Perfil Sociodemográfico de la Provincia de Lima. Tech. Rep., Instituto Nacional de Estadísticas e Informática (INEI), Lima, Peru (2008). URL `www.inei.gob.pe`.

[2] Spencer, B., Bolton, S. & Alarcon, J. The Informal Urban Communities Initiative: Community-Driven Design in the Slums of Lima, Peru. *International Journal for Service Learning in Engineering, Humanitarian Engineering and Social Entrepreneurship* **9**, 92–107 (2014).

[3] Instituto de Desarrollo Urbano-Cenca. EI Saneamiento Basico en los Barrios Marginales de Lima Metropolitana. Tech. Rep., Programa de Agua y Saneamiento PNUD-Banco Mundial, Lima.

[4] Oak Ridge National Laboratory & East View Cartographic, I. East View LandScan global 2012 (2013).

[5] Federal Emergency Management Agency (FEMA). Multi-hazard Loss Estimation Methodology: Earthquake Model. Hazus®–MH 2.1: Technical Manual. (2015).

[6] Ceferino, L., Kiremidjian, A. & Deierlein, G. Regional Multi-severity Casualty Estimation Due to Building Damage Following a Mw 8.8 Earthquake Scenario in Lima, Peru. *Earthquake Spectra* **34** (2018).

[7] Ceferino, L., Kiremidjian, A. S. & Deierlein, G. G. Probabilistic Model for Regional Multi-severity Casualty Estimation due to Building Damage Following an Earthquake. *Special Collection of ASCE-ASME Journal of Risk and Uncertainty in Engineering Systems: Part A: Civil Engineering* **4** (2018).

[8] Instituto Nacional de Estadísticas e Informática (INEI). Sistema de Consulta de la Base de Datos de los Resultados Definitivos de los Censos Nacionales 2017: XII de Población, VII de Vivienda y III de Comunidades Indígenas, (2020). URL `http://censos2017.inei.gob.pe/redatam/`.
